# *Terminalia tomentosa *Bark Ameliorates Inflammation and Arthritis in Carrageenan Induced Inflammatory Model and Freund's Adjuvant-Induced Arthritis Model in Rats

**DOI:** 10.1155/2019/7898914

**Published:** 2019-01-17

**Authors:** Srinivasa Reddy Jitta, Prasanthi Daram, Karthik Gourishetti, C. S. Misra, Picheswara Rao Polu, Abhishek Shah, C. S. Shreedhara, Madhavan Nampoothiri, Richard Lobo

**Affiliations:** ^1^Department of Pharmacognosy, Manipal College of Pharmaceutical Sciences, Manipal Academy of Higher Education, Manipal 576104, Karnataka, India; ^2^Department of Pharmacology, Manipal College of Pharmaceutical Sciences, Manipal Academy of Higher Education, Manipal 576104, Karnataka, India

## Abstract

*Terminalia tomentosa *bark belongs to the family Combretaceae. The plant bark is astringent and useful in the treatment of ulcers, vata, fractures, hemorrhages, bronchitis, and diarrhea. Phytochemical investigation of* T. tomentosa* bark confirms the presence of flavonoids, polyphenols, and tannins. The plant has not been investigated for its anti-inflammatory and antiarthritic activity. The present study was undertaken to explore its possible anti-inflammatory and antiarthritic activity. Anti-inflammatory activity of alcoholic and aqueous extracts of the bark was assessed by* in vivo* methods.* In vivo *antiarthritic potential of the extracts was evaluated by Complete Freund's Adjuvant (CFA) induced arthritis in Wistar rats. Our findings showed that the alcoholic and aqueous extracts exhibited anti-inflammatory activity at 500 mg/kg oral dose in carrageenan-induced hind paw edema and carrageenan-induced air pouch inflammation models. We also found alcoholic as well as aqueous extracts of the bark restored the altered blood and serum parameters caused by the Complete Freund's Adjuvant-induced arthritis in Wistar rats. This study shows that the* T. tomentosa* bark extracts possess anti-inflammatory activity and have pronounced effects on adjuvant arthritis also. Future studies are necessary to provide deeper insight into the exact mechanism of the action of anti-inflammatory and antiarthritic activity of* T. tomentosa*.

## 1. Introduction

Every organism eliminates foreign invaders such as infectious pathogens or damaged tissues by a complex host response known as inflammation. Inflammation is a protective response intended to eliminate the cause of cell injury. Acute inflammation is a rapid response to injuries, microbes, or other foreign substances. Chronic inflammation is a prolonged duration of inflammation in which active inflammation and tissue injuries proceed simultaneously [1]. Chronic inflammation leads to various pathological conditions like atherosclerosis, metabolic syndromes, arthritis, allergies, cancer, and various other autoimmune diseases. Currently, no substantial treatments are available for most of these conditions. General treatment relies on the use of steroidal and nonsteroidal anti-inflammatory drugs (NSAIDs) which exhibit several adverse effects [2]. Apart from NSAIDs, there are some alternative treatments available for inflammation and autoimmune diseases. Monoclonal antibodies (mAbs) are one of the treatment options giving a positive hope in the management of chronic inflammatory-related diseases. Adalimumab (trade name: Humira) is one of the human monoclonal antibodies currently available for the treatment of autoimmune diseases.

As a result of the major side effect observed with steroidal and NSAIDs and others, there is an emerging interest in natural herbal remedies and dietary supplement for treating inflammation [3]. Plant* Terminalia tomentosa *Wight & Arn. belongs to the family Combretaceae, also known as crocodile bark. The bark is known to contain carbohydrates, steroids, flavonoids, triterpenoids, tannins, and saponin. It possesses various pharmacological activities like antileucorrhoea, anti-hyperglycaemic, antioxidant, antifungal, antidiarrhoeal, etc. The bark of the plant is astringent and useful in bronchitis, ulcers, hemorrhages, vata, fractures, and diarrhea [4]. Phytochemical investigation of* T. tomentosa* bark confirms the presence of flavonoids, polyphenols, and tannins. No work has been carried out for anti-inflammatory and antiarthritic activity of* T. tomentosa *as per our knowledge. The present study was undertaken to explore its possible anti-inflammatory and antiarthritic activity [4].

## 2. Materials and Methods

### 2.1. Plant Collection

The bark of the plant was collected from Someshwara forest Hebri, Manipal, Karnataka, India (September 2014). The plant was authenticated by Dr. Gopalakrishna Bhat, Ret. Professor, Taxonomy Research Centre, Department of Botany, Poorna Prajna College Udupi, Karnataka, India. A voucher specimen (Specimen No: PP 600) was deposited in the Department of Pharmacognosy, Manipal College of Pharmaceutical Sciences, Manipal Academy of Higher Education, Manipal, Karnataka, India. The plant name was checked on the website www.theplantlist.org on 15 December 2015.

### 2.2. Preparation of the Extracts

The bark of* T. tomentosa *was dried in an air circulation oven at 37°C. It was powdered using a metallic grinder. Using the dried and powdered bark of* T. tomentosa, *an aqueous extract was prepared by maceration. For the preparation of the aqueous extract, we used a solvent system comprising chloroform and water in the ratio of 2:98 for 7 days which was subjected to occasional shaking. The prepared aqueous extract was filtered and evaporated.

Simultaneously, an alcoholic extract was prepared by subjecting the dried and powdered bark of* T. tomentosa *to Soxhlet extraction using ethanol as the solvent. The prepared alcoholic extract was filtered and evaporated by distillation. The extract was concentrated* in vacuo.* Extracts were stored at 4-5°C in a refrigerator.

### 2.3. Total Phenol Content and Total Flavonoid Content

Total phenol content of the extracts was determined according to a previously described method using Folin-Ciocalteu reagent. We used gallic acid as a standard. Total flavonoid content in the extracts was quantified as per the method described previously [5]. We used quercetin as a standard marker.

### 2.4. High-Performance Thin Layer Chromatographic Profile (HPTLC)

HPTLC was used to standardize alcoholic extract (TTE) and aqueous extract (TTW) of the bark of* T. tomentosa*. We used ellagic acid as a standard marker. The standard ellagic acid solution (100*μ*g/mL) and extracts (1mg/mL) were prepared by using HPLC grade methanol. HPTLC was performed on aluminum-backed (10 cm X 10 cm) plates coated with silica gel 60 F_254_ (Merck, Mumbai, India). The standard ellagic acid solution was prepared and extract solutions were applied on the plate in the form of bands by use of a CAMAG (Muttenz, Switzerland) Linomat-5 sample applicator equipped with a 100 *μ*L Hamilton (USA) syringe. Ascending development was performed at room temperature (25±2°C), with the mobile phase as toluene: ethyl acetate: formic acid: methanol (3:3:0.8:0.2) in a CAMAG glass twin trough chamber previously saturated with mobile phase vapor for 20 min. After development, plates were dried and then scanned densitometrically at 280 nm with a CAMAG-TLC Scanner. The amount of ellagic acid was calculated using the following formula:

%Yield= (*AUC of sample x conc. of standard *x* %purity/AUC of standard *x* conc. of sample*)

### 2.5. Experimental Animals

Male Wistar rats (180-200 g) inbred at Central Animal Research Facility, Manipal Academy of Higher Education, Manipal, were used in the study. The animal experiment protocol was approved by KMC Manipal, Institutional Animal Ethics Committee (IAEC), Manipal University, Manipal (IAEC/KMC/96/2013). Rats were housed in sterile polypropylene cages containing sterile paddy husk as bedding material. Animals were maintained at 23±3°C temperature with controlled conditions of humidity. All animals we maintained at 10/14-hour light and dark cycle. The animals were fed on autoclaved rat feed and water. The animal care and handling were carried out in accordance with guidelines issued by the Institutional Animal Ethics Committee, Manipal.

### 2.6. Acute Oral Toxicity Study

We conducted the acute oral toxicity study as per the OECD guidelines using graded doses (1000-4000 mg/kg) of TTE and TTW [6]. In brief, drugs were administered orally to Wistar rats that were fasted overnight. Animals were under general clinical observations for the first 24 hours. They were monitored closely for the first 4 hours. Thereafter, they were kept under daily observation for the duration of 14 days. We conducted Irwin's test where we observed the animals for gross behavioral changes (spontaneous motor activity, writhing, defecation, urination, pile erection, etc.) [7]. The dose selected for the extracts was 1/8th of the maximum tolerated safe dose found from acute toxicity studies.

### 2.7. Carrageenan-Induced Hind Paw Edema in Wistar Rats

Anti-inflammatory effect of TTE and TTW in* in vivo* was evaluated in Wistar rats using carrageenan-induced hind paw edema model as described earlier [8]. Carrageenan (0.1mL of 1% solution in normal saline) was injected into the plantar side of the right hind paw of the rat to induce edema. The plant extracts, i.e., TTE and TTW (500 mg/kg), diclofenac sodium (10 mg/kg), and vehicle (0.2% Na CMC), were administered orally an hour prior to the carrageenan injection to the rats. Six animals in each group (n=6) were selected after randomization based on body weight. We measured paw volume at different time points, i.e., 0, 1, 3, and 5 hr after carrageenan challenge using a digital Plethysmometer. The % increase in paw volume was calculated by using the following formula:

Percentage increase in paw volume = [(*X*-*Y*)/*Y*] × 100

In the above formula, X is paw volume at different time points after carrageenan challenge and Y is paw volume before carrageenan challenge.

### 2.8. Carrageenan-Induced Air Pouch Inflammation in Wistar Rats

As per the established protocol [8], an air pouch was created in dorsal part of rats by subcutaneously injecting 20 mL of air through a 0.22 mm filter unit on the first day. Subsequently, the patency of air pouches was maintained by injecting 10 mL air into the cavity on the third day. On the fifth day, inflammation was triggered by injecting 1 mL of carrageenan (1% w/v) into the air pouch to all groups (six animals in each group, n=6) of rats except control group. After an hour of inducing the inflammation, drug/test compounds (TTE 500 mg/kg, TTW 500 mg/kg, diclofenac sodium 10 mg/kg) were administered orally. Animals were sacrificed 6 hrs after injecting carrageenan. This was followed by giving a thorough wash to the air pouch with 5 mL of ice-cold normal saline. We estimated total leucocyte and granulocyte using a veterinary blood cell counter (PCE-210VET, Erma Inc., Tokyo, Japan). Myeloperoxidase (MPO) levels of lavage were estimated using the previously described method [9].

### 2.9. Complete Freund's Adjuvant (CFA) Induced Arthritis in Wistar Rats

Arthritis was induced by the injection of 0.1 mL of Complete Freund's Adjuvant, CFA (heat-killed* Mycobacterium butyricum* in paraffin oil, 5.0 mg/mL) into the subplantar region of the left hind paw. After an initial measurement of paw volumes, we divided the rodents into different groups (six animals in each group, n=6) on the seventh day. They were classified into CFA control (positive control), diclofenac (10mg/kg, p.o.), TTE (500mg/kg, p.o.), and TTW (500mg/kg, p.o.). Dosing was carried out from the fourteenth day to the twenty-first day.

### 2.10. Evaluation of Severity of Arthritis

Paw volumes of both ipsilateral (CFA injected paw) and contralateral (CFA noninjected paw) paws were measured on the 7th, 14th, and 21st day following the CFA injection. The animals were demarcated at the level of the lateral malleolus and measured by digital Plethysmometer. The severity of arthritis of each paw was scored as per the scoring system described previously [10]. Briefly, the intensity of arthritis was scored by grading each paw from 0 to 4 based on erythema, swelling, and deformity of the joint (scores were defined as 0 = no erythema or swelling; 1 = slight erythema or swelling of one of the toes or fingers; 2 = erythema and swelling of more than one toe or finger; 3 = erythema and swelling of the ankle or wrist; 4 = complete erythema and swelling of toes or fingers and ankle or wrist, and inability to bend the ankle or wrist). A researcher who was not involved in the study was familiarized with the scoring system. The researcher assessed and evaluated the severity and gave suitable scores. On the 21st day, blood was collected using the retro-orbital puncture method and hematological parameters were evaluated using a cell counter (PCE-210VET, Erma Inc., Tokyo, Japan). The levels of serum C-reactive protein and rheumatoid factor were estimated using commercially available ELISA kits (MyBiosource, San Diego, California, USA). The calcium level of serum was estimated with an autoanalyzer.

### 2.11. Radiological and Histopathological Analysis

On the 21st day, images of the hind paws were taken using X-ray (the exposure time was 0.08s, the film focus distance was 45 inches, and the machine was operated at 60 kV peak, 8 mA) under anesthesia and evaluated for its radiographic changes. At the end of the experiment (22nd day) animals were sacrificed by asphyxiation using carbon dioxide. The brain, spleen, and thymus of all the animals were removed and weighed. The amputated hind paw ankles were fixed in 10% neutral-buffered formalin, decalcified in 10% formic acid, dehydrated, and then processed and embedded in paraffin. The 5 *μ* sections were stained with hematoxylin and eosin and evaluated in a blinded manner. Cellular infiltration, synovial hyperplasia, pannus formation, and bone and cartilage erosion of the ankle joints were evaluated histopathologically [11].

### 2.12. Statistical Analysis

Results are expressed as mean ± SEM and analyzed statistically using one-way ANOVA with Dunnett's post hock test. This was performed using GraphPad Prism version 5.00 for Windows, GraphPad Software, San Diego, California, USA. P < 0.05 was considered as statistically significant.

## 3. Results

### 3.1. Total Phenol, Total Flavonoid Content, and HPTLC Profiling

**Figure 1:**
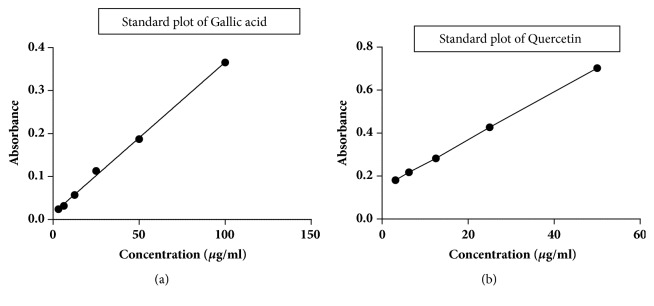
(a) Standard plot of gallic acid. (b) Standard plot of quercetin.

**Figure 2:**
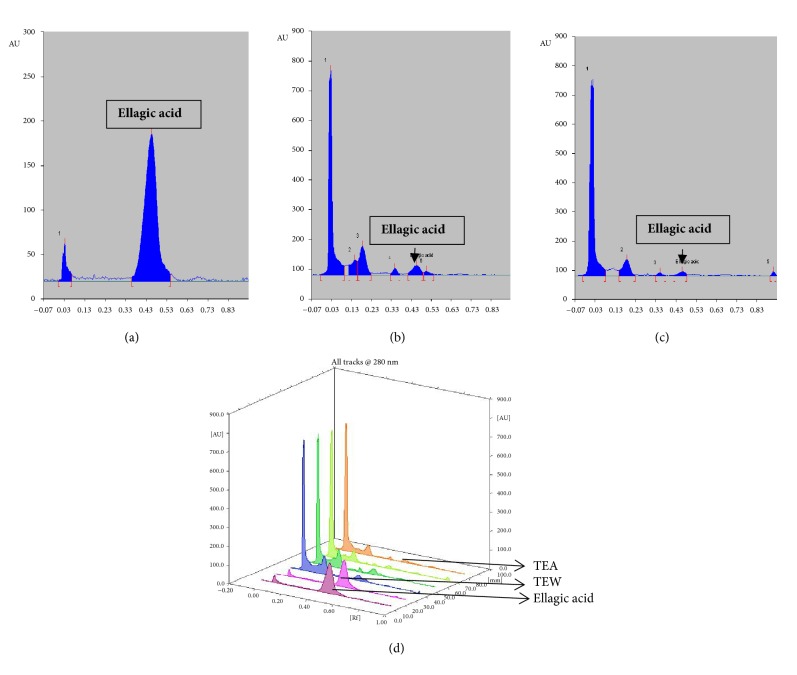
HPTLC profile and chromatograms: (a) ellagic acid; (b) TTE; (c) TTW; (d) chromatogram of ellagic acid, TTE, and TTW.

The total phenolic content of ethanolic and aqueous extracts of* T. tomentosa* bark was found to be 457 mg/g and 352 mg/g of GAE (gallic acid equivalents), respectively, as depicted in Figure 1(a). The total flavonoid content of ethanolic and aqueous extracts of* T. tomentosa* was found to be 12.53 mg/g and 21 mg/g of QE (quercetin equivalents), respectively, as depicted in Figure 1(b). The tannin content of the stem powder was found to be 5.8% w/w. The ellagic acid content of the alcoholic (TTE) and aqueous (TTW) extracts was found to be 0.712% w/w and 0.25% w/w, respectively.  Figure 2 represents the HPTLC profile and chromatograms of TTE, TTW, and ellagic acid.

### 3.2. Acute Toxicity Studies

After 14 days of observation followed by dosing maximum dose of 3500 mg/kg, we found no significant toxic signs such as spontaneous motor activity, writhing, defecation, urination, and pile erection.

### 3.3. Carrageenan-Induced Hind Paw Edema in Wistar Rats

**Figure 3:**
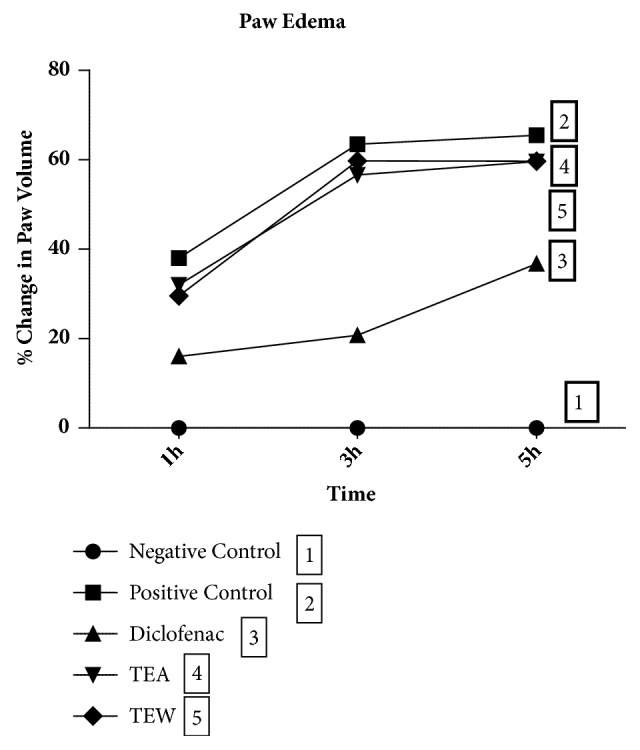
Carrageenan-induced hind paw edema in Wistar rats, diclofenac sodium used as a standard drug and compared with the effect of ethanolic and water extracts of* T. tomentosa*.

We observed acute hind paw inflammation in rats following an intraplantar carrageenan injection. Diclofenac significantly reduced the paw edema at 1st, 3rd, and 5th hour (0.15±0.0 cm, 0.20±0.0 cm, and 0.35±0.1 cm) compared to the carrageenan control (0.41±0.1 cm, 0.69±0.1 cm, and 0.70±0.1 cm). TTE and TTW reduced the paw edema in the first hour (0.32±0.1 cm and 0.30±0.0 cm) moderately. Percentage changes in paw volume of rats with diclofenac sodium, TTE, and TTW treatments are shown in Figure 3.

### 3.4. Carrageenan-Induced Air Pouch Inflammation in Wistar Rats

**Figure 4:**
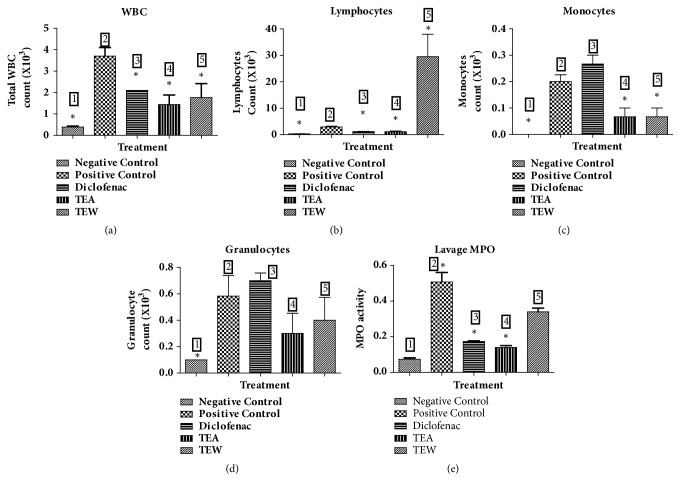
Carrageenan-induced air pouch inflammation in Wistar rats: (a) total WBC count, (b) % lymphocytes count, (c) % granulocytes count, (d) % monocytes count, and (e) MPO activity. 1: negative control, 2: positive control, 3: diclofenac, 4: TEA, and 5: TEW.

We observed that diclofenac significantly reduced total WBC, lymphocyte, and MPO but failed to decrease granulocyte and monocytes count. TTE and TTW at an oral dose of 500 mg/kg significantly reduced total WBC, lymphocyte, monocyte, granulocyte, and MPO activity in air pouch lavage when compared with those of the positive control, as depicted in Figure 4.

### 3.5. Complete Freund's Adjuvant-Induced Arthritis in Wistar Rats

**Figure 5:**
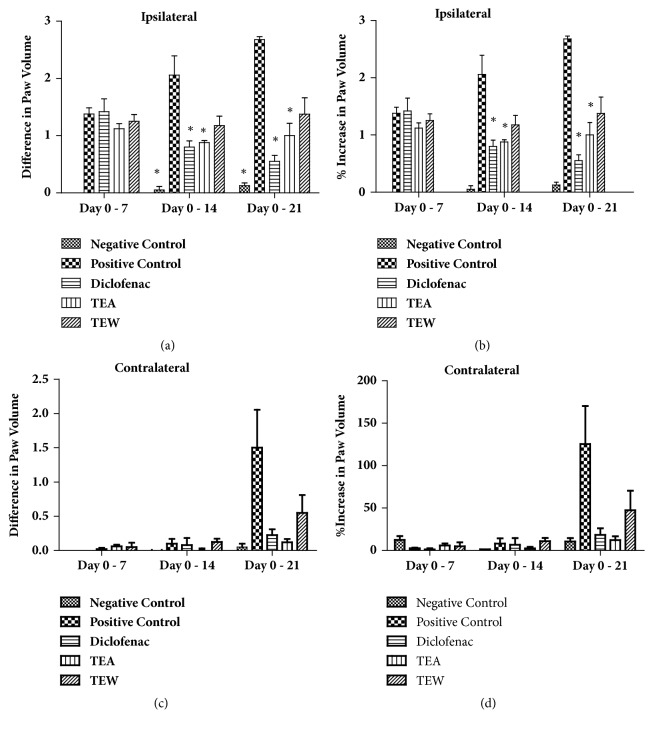
Complete Freund's Adjuvant-induced arthritis in Wistar rats. Diclofenac sodium was used as a standard drug and the effect of ethanolic and water extracts of* T. tomentosa *was compared with disease control.

Paw inflammation was observed to be significantly high in ipsilateral and contralateral paw in CFA-induced control rats. TTE and TTW reduced the % increase in paw volume in the ipsilateral and contralateral paws significantly when compared to positive control. This is shown in Figure 5.

### 3.6. Assessment of Severity of Arthritis

#### 3.6.1. Effect on Organ Weights

TTE and TTW failed to reduce the spleen weight (0.89±0.08 g, 0.76±0.05 g, and 0.95±0.08 g) when compared to positive control (0.68±0.10 g). TTE, TTW, and diclofenac reduced thymus and brain weight ratio (0.10±0.10, 0.05±0.0, and 0.07±0.0) as compared to the positive control (0.25±0.1). We also observed that TTE and TTW improved the body weight (3.6±1.5g and 3.4±2.5g) when compared to positive control (decreased by 2±0.7g) whereas diclofenac failed (decreased by 1.2±1.8 g).

#### 3.6.2. Effect on Hematological Parameters, Serum Calcium, RF, and C-RP

TTE and TTW improved RBC count but failed in decreasing WBC count whereas diclofenac failed to improve RBC as well as WBC count.  Table 2 represents the effect of TTE, TTW, and diclofenac on RBCs and WBCs. We found that TTE, TTW, and diclofenac decreased the serum calcium and RF levels when compared to positive control but failed to decrease C-RP levels as shown in Table 1.

#### 3.6.3. Effect on the Arthritic Score

Treatment with TTE, TTW, and diclofenac improved arthritic scores when compared to positive control as shown in Table 2.

#### 3.6.4. Effect on Radiological Changes

**Figure 6:**
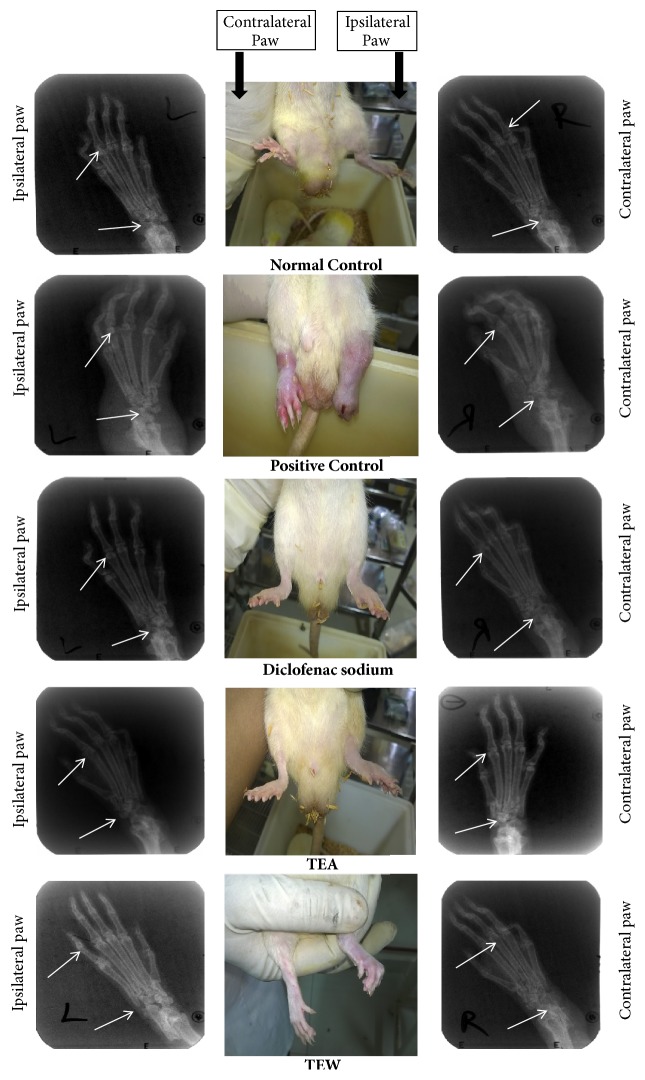
Effect on radiological changes; diclofenac sodium was used as a standard drug and the effect of ethanolic and water extracts of* T. tomentosa* was compared with disease control, where the plant extract significantly reduced the inflammation in the treatment group.

**Figure 7:**
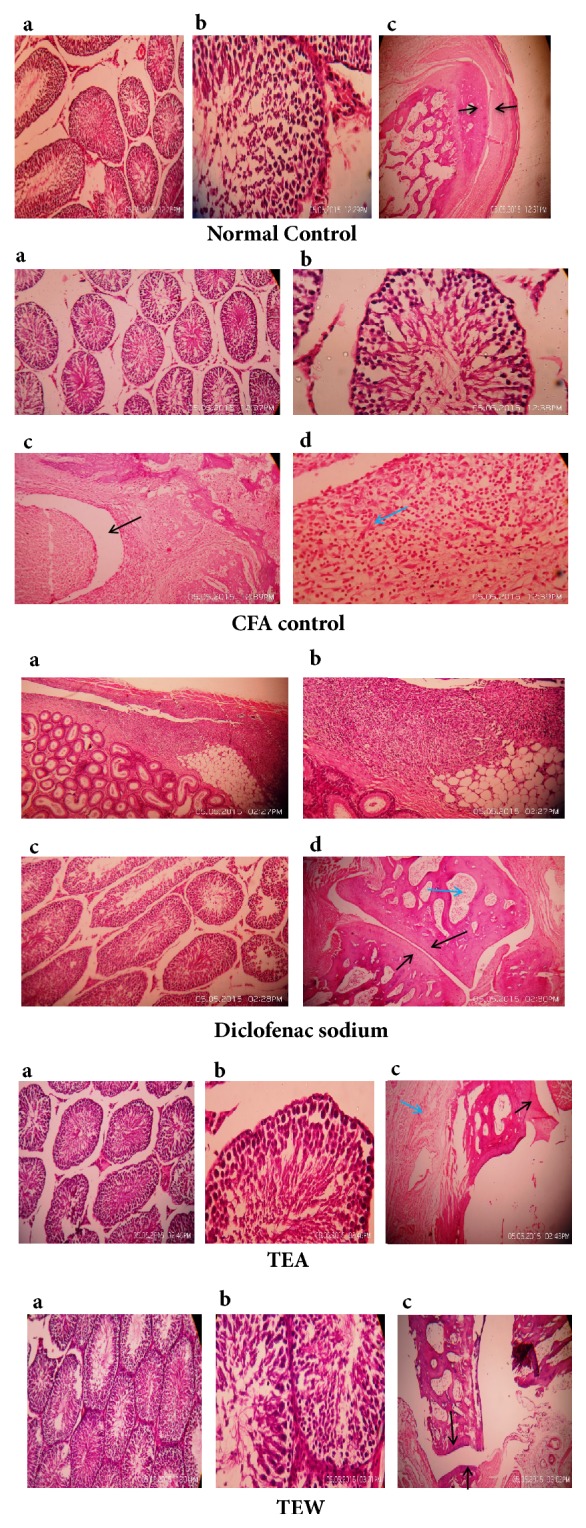
Effect on histopathological changes.

The radiographic and normal pictures of hind paws of rats with different treatments on the 21st day of the study are shown in Figures 6 and 7.

#### 3.6.5. Effect on Histopathological Changes

TTE (500 mg/kg), TTW (500 mg/kg), and diclofenac treated rats showed a lower level of soft tissue swelling, cystic enlargement of bone, extensive erosion, and bone destruction as seen from the radiographic impression and histopathological study. The differences were substantial when compared to CFA control. The efficacy of TTE and TTW is comparable to diclofenac as seen in Figure 7.

## 4. Discussion

The present study was carried out to investigate the acute and chronic anti-inflammatory activity of* T. tomentosa *Wight & Arn. plant bark in* in vivo* models. Inflammation constitutes a highly coordinated set of events which allows the tissues to respond against injury. The mechanism of action of NSAIDs is still obscure, but most of the NSAIDs interact with plasma proteins. Most of the NSAIDs stabilize serum albumin and also other fractions against heat coagulation. NSAIDs at low concentration do not alter the conformation of proteins directly and also do not inhibit the specific combination of proteins. But they influence the conformational changes affected by some proteins on heating. This suggests that NSAIDs interact with proteins in some way or another [12].

TTE and TTW may be attenuating the effects of serotonin and histamine release during acute inflammation as both extracts significantly lowered paw edema in the carrageenan-induced inflammation model during the first phase of edema development but failed to reduce it in the second phase. Edema depends on the participation of polymorphonuclear leukocytes and kinins with their proinflammatory factors including prostaglandins. The development of edema is biphasic. During the initial phase serotonin and histamine get released and in the second phase, prostaglandin-like substances are released which accelerates the process of swelling. The second phase of edema is useful to both clinically useful steroidal and nonsteroidal anti-inflammatory agents [13].

In carrageenan-induced air pouch inflammation, TTE and TTW significantly inhibited cellular infiltration such as neutrophils and granulocytes into the air pouch fluid (showing primary immunological response) and also myeloperoxidase, from the neutrophil's azurophilic granules which is responsible for invoking tissue injury. TTE and TTW have considerable potential as a therapeutic inhibitor of MPO-mediated tissue damage [8].

Rat adjuvant-induced arthritis is the commonly used animal model for the studies of NSAIDs and disease modifying antirheumatic drugs. Development of arthritis can be divided into three phases. It starts with the induction phase without any evidence of synovitis, followed by early synovitis and finally late synovitis with progressive joint destruction. An effective antirheumatic agent should be able to block one or more of these three phases [13, 14] whereas both the extracts were able to suppress the joint inflammation and synovitis. It also showed very effective prevention of systemic inflammation which ultimately reduced the destruction of joints as seen in the arthritic scores and pictures as represented in Figure 6.

TTE and TTW exhibited antiarthritic effect by improving the body weight of treated animals as compared to positive control animals. TTE and TTW treatment reduced the serum levels of rheumatoid factor (RF) significantly and RF is one of the well-known strong indicators of rheumatoid arthritis.

CFA induced arthritis in rats is speculated to happen through cell-mediated autoimmunity and is because of the structural mimicry between cartilage proteoglycans and* Mycobacterium* in rats [15]. The development of secondary lesions on contralateral paw (i.e., % increase in paw volume of noninjected paw) after 7th day after adjuvant injection is an indication of cell-mediated immunity. TTE and TTW reduced the secondary lesion. This suggests its immunomodulatory effect and demonstrates its efficacy in the suppression of cellular immunity [8]. Both the extracts decreased the arthritic clinical scores of all the paws and reduced secondary arthritic lesions. This finding further distinguishes its immunosuppressive effects from its anti-inflammatory effects.

Reduced bone formation and increased bone resorption are a major cause of bone destruction in adjuvant-induced arthritis in rats. In this regard, the radiographic images may give a clear understanding of disease status and its remission. The radiographic pictures clarified the decreased bone loss in treatment groups in comparison to the arthritic group. It has been suggested that new therapeutic strategies for chronic forms of arthritis treatment have to aim at both joint protection and suppression of synovitis. These are known to be the ultimate goals of an effective arthritis treatment. TTE and TTW decreased the serum calcium levels when compared to disease control animals which are an indicator of reduced bone resorption [13].

The synovial fluid is enriched predominantly with neutrophils. Aggressive macrophages, T lymphocytes, dendritic cells, and activated fibroblasts are a general phenomenon in humans with arthritis. Hence, adjuvant-induced arthritis has been used extensively for pharmaceutical testing. Therefore, data is widely available for comparison with human clinical trials. The junction between the synovial membrane lining the joint capsule and the cartilage (with a mass rich in macrophages) often termed the pannus is a primary site of irreversible tissue damage. The cells of the pannus migrate over the underlying cartilage and into the subchondral bone. This may lead to subsequent erosion of tissues. The activated lymphocytes, macrophages, and fibroblasts and their products can also stimulate angiogenesis, which may result in increased vascularity in the synovium of patients with rheumatoid arthritis.

Histopathology studies show pannus formation in positive control (CFA control) animals which indicates joint destruction. It was absent in the treatment groups (TTE, TTW, and diclofenac). Joint destruction was not prominent in treatment groups as compared to the positive control group. In late disease stages, the pannus (membrane from granulation tissue, which is chronic and progressive and produces joint erosion) extends over the surface of articular cartilage, resulting in fibrous ankylosis. We did not observe these in our treatment groups.

Plant extracts and a mixture of tannins showed apparent anti-inflammatory activity in carrageenan-induced acute inflammation and adjuvant-induced chronic inflammation (polyarthritis) in rats. Tannins might produce effects in a nonspecific manner due to their astringent properties on cell membranes thus affecting cell functions [14]. The above results suggest that polyphenols may have played a critical role in the anti-inflammatory activity, as polyphenols present in the herbs are known to inhibit both IU and TNF-*α*, and stimulated IL-8 release from cells [16]. Flavonoids are known to have different biological roles. The anti-inflammatory activity of flavonoids in* in vitro* or in cellular models may cause inhibition of the synthesis of different proinflammatory mediators (cytokines, eicosanoids, adhesion molecules, and C-reactive protein). However, the mechanism of action was not identified clearly [17]. The preliminary phytochemical studies carried out on the extracts showed the presence of tannins, flavonoids, polyphenols, carbohydrates, and saponins which have a major role in reducing inflammation. HPTLC profiling of extracts shows the presence of ellagic acid and it provides suitable standards for the identification of this plant material for future investigation. Evidence suggests that ellagic acid interferes with the function of PMNs by inhibiting the release of MPO, thus showing its anti-inflammatory activity [8]. Many of the natural compounds inhibit the inflammatory pathways in a similar way to NSAIDs. Even though the mechanism of action is not clear for most of the anti-inflammatory drugs, it is evident that Adalimumab monoclonal antibodies suppress TNF-*α* expression. TNF-*α* is one of the major contributors to inflammation in autoimmune diseases. In addition to the COX inhibitory pathway, many natural compounds inhibit nuclear factor-*κ*B (NF-*κ*B) cascade [3]. The exact mode of action involved in acute and chronic anti-inflammatory effects of TTE and TTW is not known. We hypothesize that one or more of the plant metabolites could be responsible for its pharmacological effects. Our studies have shown that the bark extract of* T. tomentosa* possesses effective edema-suppression, neutrophil infiltration, and antiarthritic property with minimal toxicity thus playing a promising role in the treatment of acute and chronic inflammation. Further studies on* T. tomentosa* are necessary to reveal the exact mechanism of actions.

## 5. Conclusion

To conclude,* T. tomentosa *possesses significant anti-inflammatory activity and has pronounced effects on adjuvant arthritis as well. Future studies are necessary to provide deeper insight into the exact mechanism of action of anti-inflammatory and antiarthritic activity of* T. tomentosa*.

## Figures and Tables

**Table 1:** Alterations in hematological, serum parameters in CFA induced arthritis in Wistar rats. Results expressed as mean ± SEM (n = 6) analyzed by one-way ANOVA followed by Dunnett's post hoc test, ^*∗*^p < 0.05 as compared with positive control [RBC: red blood cell; Hb: hemoglobin; WBC: white blood cell; CRP: C-reactive protein; RF: rheumatoid factor].

Treatment	Hematological parameters			Serum parameters		
	RBC (10^6^/*µ*L)	Hb (g/dL)	WBC (10^3^/*µ*L)	C-RP (IU/mL)	RF (IU/mL)	Calcium (mg/dL)
Positive control	8.82±0.49	11.32±0.66	10.36±1.25	0.32±0.00	0.28±0.00	11.4±0.2
Diclofenac sodium (10mg/kg)	7.46±0.21^*∗*^	9.00±0.35^*∗*^	15.55±3.03^*∗*^	0.75±0.02^*∗*^	0.17±0.05	10.5±0.2
TTE	9.99±0.39	11.96±0.50	10.30±1.15	0.78±0.25^*∗*^	0.16±0.09	10.9±0.2
TTW	9.54±0.25	11.38±0.42	11.38±1.67	0.72±0.13^*∗*^	0.17±0.07	11.0±0.2

**Table 2:** Effect of TTE, TTW, and diclofenac on arthritic clinical scoring.

Sl. No	Treatment	Clinical scoring					
		Day 0	Day7	Day 10	Day 14	Day 17	Day 21
**1**	Positive Control	0	1	2	2	3	3
**2**	diclofenac sodium	0	1	1	2	2	2
**3**	TTE	0	1	1	2	2	2
**4**	TTW	0	1	1	1	2	2

## Data Availability

The data used to support the findings of this study are available from the corresponding author upon request.

## Abbreviations

%: Percent
°C: Celsius
*μ*g: Microgram
*μ*l: Microliter
ANOVA: Analysis of variance
AUC: Area under Curve
CFA: Complete Freund's Adjuvant
cm: Centimeter
Conc.: Concentration
C-RP: C-reactive protein
DMSO: Dimethyl sulfoxide
ELISA: Enzyme Linked Immunosorbent Assay
g: Grams
Hb: Haemoglobin
H_2_O_2_: Hydrogen peroxide
HPTLC: High Performance Thin Layer Chromatography
IC_50_: Concentration yielding 50% inhibition
IU: International units
Kg: Kilogram
mg: Milligram
min: Minutes
ml: Milliliter
MPO: Myeloperoxidase
nm: Nanometers
NSAIDs: Nonsteroidal anti-inflammatory drugs
P.O: * per os* (by mouth)
RBC: Red blood cell
R_f_: Retention factor
RF: Rheumatoid factor
RPM: Rotations per minute
SEM: Standard error of mean
UV: Ultra violet
*T. tomentosa:*: * Terminalia tomentosa*
TTE: * Terminalia tomentosa* ethanolic extract
TTW: * Terminalia tomentosa* water extract
V/V: Volume by volume
W/V: Weight by volume
W/W: Weight by weight
WBC: White blood cell.
